# Bridging lab and field: *in situ* oxygen uptake measurements match laboratory-derived values in *Sebastes fasciatus*

**DOI:** 10.1093/conphys/coag090

**Published:** 2026-11-12

**Authors:** Joëlle J Guitard, Denis Chabot, Dominique Robert, David Deslauriers

**Affiliations:** Institut des sciences de la mer, Université du Québec à Rimouski, 310 All. des Ursulines, Rimouski, QC, G5L 2Z9, Canada; Maurice Lamontagne Institute, Fisheries and Oceans Canada, 850 route de la mer, Mont-Joli, QC, G5H 3Z4, Canada; Institut des sciences de la mer, Université du Québec à Rimouski, 310 All. des Ursulines, Rimouski, QC, G5L 2Z9, Canada; Maurice Lamontagne Institute, Fisheries and Oceans Canada, 850 route de la mer, Mont-Joli, QC, G5H 3Z4, Canada

**Keywords:** Autonomous instrumentation, environmental variability, field respirometry, intermittent-flow respirometry, metabolic rates

## Abstract

Under laboratory conditions, measuring oxygen uptake provides a reliable proxy for metabolic rate. However, this requires that fish be captured and transported to the laboratory, which may influence their physiology. In addition, laboratory conditions may differ from the wild rendering the tests less relevant for conservation research. The objective of the present study was to develop, describe and evaluate a minimally invasive respirometry approach for estimating metabolic rates of fish *in situ*. Two custom-built respirometers were deployed at a depth of 30 m in the St. Lawrence Estuary (Canada) to measure oxygen uptake in wild redfish (*Sebastes fasciatus*) over a 24-hour period (*n* $\boldsymbol{=}$ 10). Of these, four individuals were subsequently re-sampled in the laboratory to compare *in situ* routine metabolic rate (RMR) with laboratory standard metabolic rate (SMR). Additional SMR measurements were obtained from laboratory-acclimated S. fasciatus (*n* $\boldsymbol{=}$ 11). Despite natural fluctuations in temperature, light, gut fullness and potential biotic stimuli, RMR measured in the wild was consistently similar to laboratory-derived SMR. These findings demonstrate the feasibility of *in situ* respirometry for estimating oxygen uptake while maintaining ecological relevance.

## Abbreviations

AS, Aerobic scope
DO, Dissolved oxygen
HOBO, HOBO dissolved oxygen logger (commercial logger brand/model)
IQR, Interquartile range
ISMeR, *In situ* respirometry
Lab-Acclim, Laboratory respirometry on acclimated fish
Lab-ISMeR, Laboratory respirometry on fish previously used in ISMeR
MMR, Maximum metabolic rate
PIT-tag, Passive Integrated Transponder tag
*P*O_2_, Partial pressure of oxygen
RMR, Routine metabolic rate
SMR, Standard metabolic rate
*Ṁ*O_2_, Oxygen uptake rate

## Introduction

Metabolic rate is a fundamental ecological process, as it governs energy assimilation, transformation and allocation, directly shaping key functions for maintenance, resource use, growth and reproduction (Brown *et al.*, 2004). Estimating metabolic rates through oxygen uptake in fishes provides insight on the potential energy available to an organism and can be achieved using well-established respirometry techniques (Steffensen, 1989; Svendsen *et al.*, 2016; Killen *et al.*, 2021). However, determining meaningful levels of metabolic rate from the collected data can be more challenging. Standard metabolic rate in fishes (SMR: the minimal metabolic rate needed to sustain life in a resting, fasting and non-stressed condition) is ecologically significant because it provides a basis for determining aerobic scope (AS)—the energy available for activities like movement, digestion and reproduction, beyond basal metabolic needs (Claireaux and Lefrançois, 2007; Chabot *et al.*, 2016). SMR also constitutes a valuable input into bioenergetic models predicting energy allocation. To estimate SMR, the fish must be in a post-absorptive state and exhibit minimal activity or movement. Even though most of these measurements are obtained in the laboratory, such strict conditions cannot always be met, and the resulting oxygen uptake is then referred to as routine metabolic rate (RMR). RMR includes the small, spontaneous activity that fish typically display in a respirometer—such as maintaining position or occasional fin movements, and therefore represents a broader, less strictly defined measure of baseline metabolism (Chabot *et al.*, 2016). Because activity is not quantified, RMR can range from SMR to substantially higher values. However, appropriate respirometer design generally minimizes activity, keeping RMR close to SMR (Chabot *et al.*, 2016). This variability in the literature reflects the reality that RMR is often the most practical metric for studies in which true resting conditions cannot be confirmed, particularly under field conditions where spontaneous activity cannot be completely eliminated.

Most respirometry studies are conducted in controlled laboratory environments, where animals are either bred in captivity or transported from their natural habitats. As mentioned by Turko *et al.* (2023), laboratory animals can differ physiologically from wild animals, due to differences in diet, parasite load and stress exposure. In addition, bringing animals in the laboratory often involves various interventions, such as antiparasitic measures and rearing in conditions that do not fully reflect their natural environment (Turko *et al.*, 2023). These conditions may alter physiological phenotypes and result in the loss of individuals that do not adjust well to captivity, meaning that the animals remaining in experiments may not fully express the physiological diversity found in the wild. Consistent with these concerns, recently captured wild sunfish had greater hypoxia tolerance and resting *Ṁ*O_2_ values that were similar to or lower than those of laboratory-acclimated fish (Borowiec *et al.*, 2016).

Studying fishes in the lab can also lead to overestimation or underestimation of certain metabolic traits. For example, maintaining fishes at constant temperatures in the lab may not be relevant if wild individuals live where temperature fluctuates daily. Beauregard *et al.* (2013) showed that Atlantic salmon (*Salmo salar*) exposed to circadian fluctuations in water temperature had SMRs 25–32% higher than conspecifics kept at a constant temperature, suggesting that standard lab protocols may underestimate metabolic rates of fish from variable natural environments. These findings highlight the importance of accurate metabolic measurements obtained in environmental conditions relevant for the target species, particularly when such data are used in bioenergetic models intended to simulate growth under natural, fluctuating conditions.

While minimizing stressors in laboratory experiments can improve the precision of metabolic rate estimates (Auer *et al.*, 2016), measurements taken directly in natural habitats could offer more ecologically relevant insight into physiological responses, which would thus be highly valuable for developing accurate models. Few studies have estimated metabolic rates in natural environments due to logistical constraints, however those that did were driven by ecological and conservation objectives, leading to different methodological approaches. For instance, Farrell *et al.* (2003) deployed a mobile streamside swim tunnel to estimate the oxygen uptake and swimming capacity of migrating Pacific salmon (*Oncorhynchu*s spp.), including the energetic challenges of upstream migration. Another example is the streamside experiment on desert Redband trout (*Oncorhynchus mykiss* ssp.) testing for physiological adaptations to variability in seasonal flow and diel water temperature (Gamperl *et al.*, 2002). More recently, field-based respirometry has been used to assess seasonal variation in metabolic rate and hypoxia tolerance in the threatened eastern sand darter (*Ammocrypta pellucida*) and to compare physiological tolerances between the threatened Pugnose shiner (*Miniellus anogenus*) and a non-imperilled congener (Firth *et al.*, 2023; Reemeyer *et al.*, 2024). Together, these studies demonstrate the value of assessing oxygen uptake and other physiological traits under conditions that reflect the ecological challenges experienced by the species, thereby linking physiology more directly to conservation and management questions.

Other studies have designed automated deep-sea respirometers for species that cannot survive being brought to the surface due to barotrauma (Drazen and Seibel, 2007; Drazen and Yeh, 2012). While these systems have provided metabolic data for previously unstudied species, they do not allow control over which individuals enter the respirometer. Additionally, some field respirometry setups have been adapted for remote locations without electricity and rely only on the natural current of the stream to flush the respirometer where the experiment is taking place (Mochnacz *et al.*, 2017). These methodological advancements have improved data collection for metabolic and conservation research but still involve substantial logistical requirements, including transporting specialized equipment, deploying sophisticated underwater systems, and, in some cases, relying on natural water flow rather than pumps in intermittent-flow respirometry (Farrell *et al.*, 2003; Drazen and Yeh, 2012; Mochnacz *et al.*, 2017).

In the present study, we aimed to build and use a simple and autonomous *in situ* respirometer to estimate metabolic rates of wild fish in their natural environment using Acadian redfish (*Sebastes fasciatus*) as a model. *Sebastes fasciatus* represents a particularly relevant species for testing this approach because its physoclistous swim bladder makes capture from depth and transport to laboratory facilities challenging, requiring gradual decompression procedures to minimize barotrauma (Laroque, 2000). At the same time, its relatively sedentary behaviour and tolerance of close approach by divers allow individuals to be captured underwater with dip nets with limited pursuit, making the species well suited for *in situ* respirometry (ISMeR) trials. An *in situ* approach therefore provides an opportunity to obtain physiological measurements while reducing the need for prolonged decompression and transport to laboratory facilities. As a commercially important species in eastern Canada, obtaining additional physiological information under natural conditions is also relevant to its management (Senay *et al.*, 2026). The first objective was to describe the design and application of the system and evaluate its feasibility for obtaining reliable oxygen uptake (*Ṁ*O_2_) measurements under field conditions while minimizing handling and transport of fish. The second objective was to assess the resulting metabolic rate estimates by comparing measurements obtained *in situ* with those obtained under controlled laboratory conditions, both for the same individuals and for other *S. fasciatus* of similar size. Because ISMeR trials involve greater environmental variability, shorter acclimation and measurement periods, and an inability to assess whether fish are fully post-absorptive, we predicted that metabolic rates measured *in situ* would exceed laboratory-derived SMR values.

## Materials and methods

### Study site, sampling and collection

*Sebastes fasciatus* were sampled or captured near Les Escoumins, Québec, Canada (48.317801°N, 69.413287°W) in September–November 2019, November 2021 and September 2022. The collection site was at a depth of 25–30 m accessible to SCUBA divers. A subset of *S. fasciatus* underwent an ISMeR trial that lasted ca. 24 h (see *In situ* respirometry trials section). *Sebastes fasciatus* that underwent ISMeR and other collected *S. fasciatus* (ca. 800 in 2019, 200 in 2021 and 100 in 2022) were successfully brought live to our aquaculture facilities using the methodology developed by Laroque (2000) and successfully used in (Guitard *et al.*, 2025, 2026). Briefly, SCUBA divers caught *S. fasciatus* with dip nets and placed them into cages (33 × 34 × 23 cm) that could hold up to 30 individuals. Most fish measured between 16 and 20 cm at the time of capture. Once full, cages were placed higher up along the slope, at a depth of 10–15 m depending on the tide, to reduce barotrauma. The duration of this equilibration period was at least 12 h, but up to 96 h for fish caught early during each of the 5-day fishing trips. The day after the last capture, the cages were brought to the surface for transportation in aerated tanks to the Maurice Lamontagne Institute in Mont-Joli, Québec, Canada (48.580002°N, 68.180000°W). Fish collection in their natural habitat was carried out under a Canada Parks permit (SAGMP-2019-33741) and was approved by the Animal Care Committees of the Maurice Lamontagne Institute (Certificate 19-7 and 21-3B). The experimental methods comply with the regulations of the Canadian Council on Animal Care and were approved by the Animal Care Committee of the Maurice Lamontagne Institute (Certificate 19-6C) (Fig. 1).

**Figure 1:**
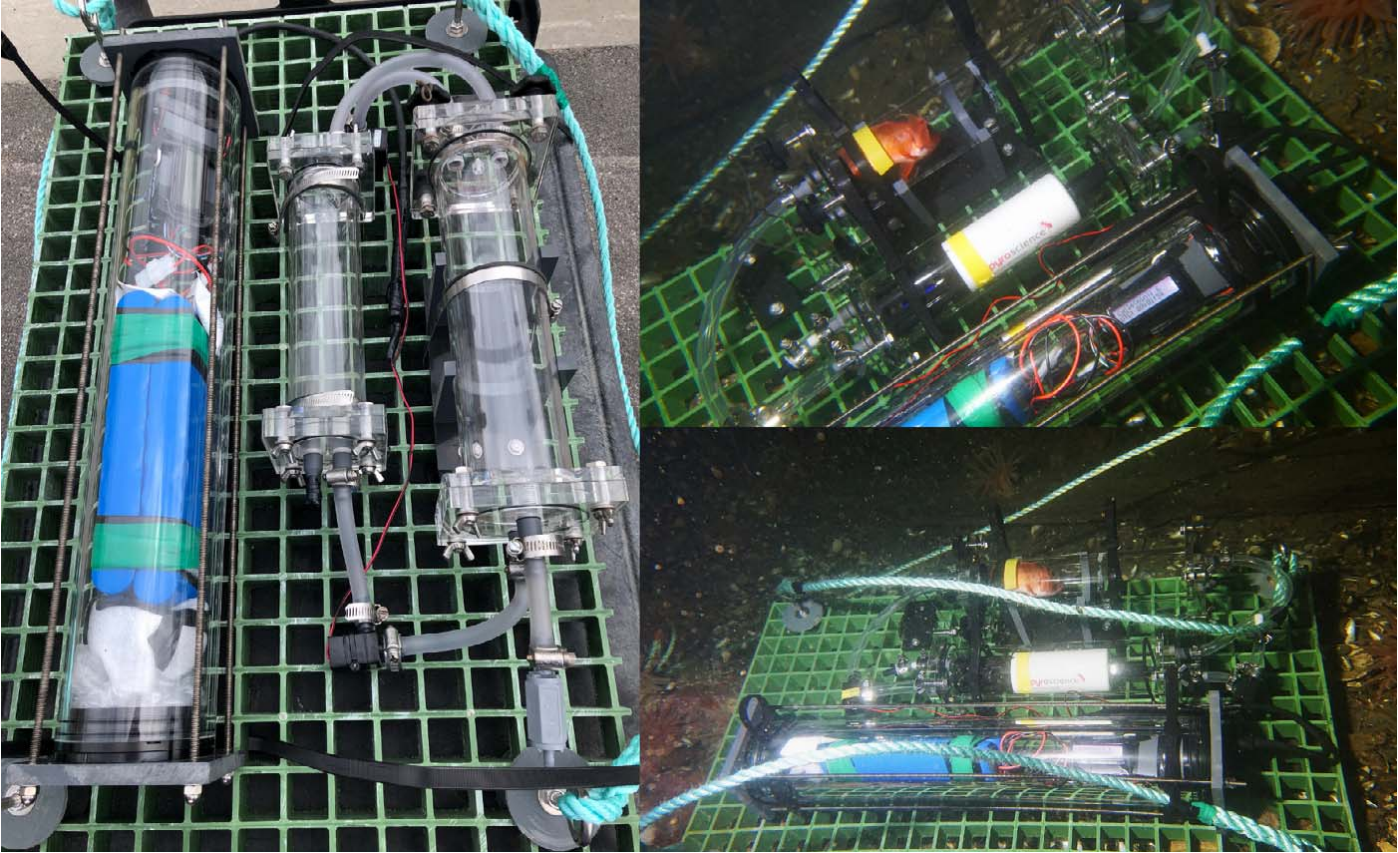
Picture of the 2021 assembled setup outside of water (left). Pictures of the 2022 setup in use underwater (right). Also see Figure S2 and Table S1 for details of the setup assembly.

Upon their arrival, fish were transferred into circular water tanks (1 m height × 1.1 m diameter; 760 l) supplied with 5°C oxygenated seawater (salinity of 28 ppt). Temperature and dissolved oxygen (DO) conditions recorded at the collection site during fish collection and the *in situ* trials are presented in Table 2 and Fig. 2. Fish that underwent respirometry trials were kept in separated tanks from the other fish brought to our facilities for our collection. All fish were offered food (northern shrimp, *Pandalus borealis*, capelin, *Mallotus villosus*, and krill, *Euphausia superba*) three times a week. It took about two weeks for the individuals to acclimatize to their new environment and feed correctly. Wild fish may present various levels of parasite load, which are known to influence performance traits (Guitard *et al.*, 2022; Chrétien *et al.*, 2023). Hence, a week after they started eating, fish received a formaldehyde treatment (37% formaldehyde, 0.01 ml L^−1^) for 1 hour to remove possible external parasites. To minimize stress, this preventive treatment was not applied to fish used for ISMeR before they underwent laboratory respirometry. One month after they started eating, fish were anaesthetized (benzocaine, 0.5 ml of solution per litre of seawater, solution = 100 g of benzocaine dissolved in 1 l of 70% ethanol), weighed, measured and individually identified with a Passive Integrated Transponder tag (PIT-tag; Biomark, Idaho, USA).

**Figure 2:**
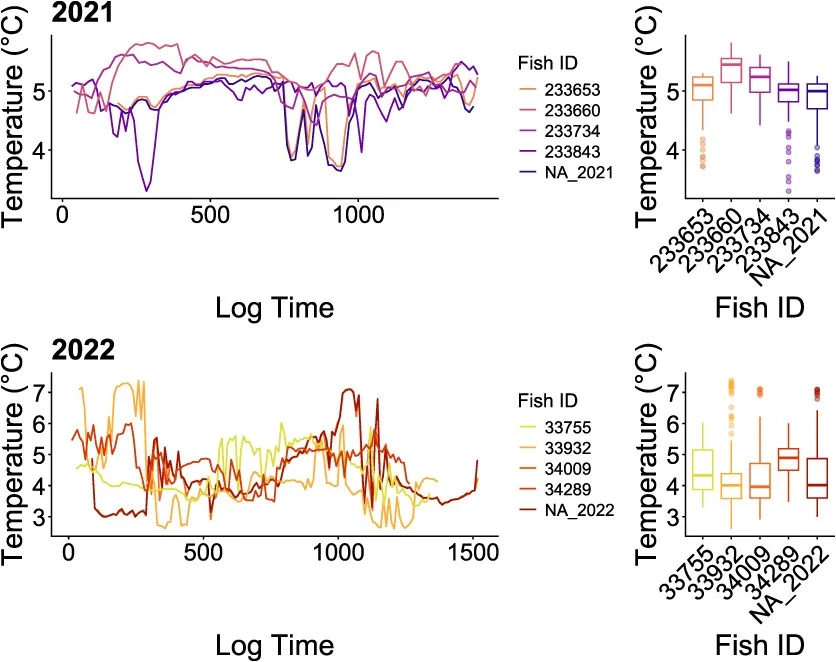
Temperature recorded during ISMeR trials in 2021 (top panel) and 2022 (bottom panel) for redfish (*Sebastes fasciatus*). Each line represents an individual fish, identified by its own colour.

### Layout of experiments

This study generated three sets of respirometry data: (i) ISMeR, (ii) controlled laboratory respirometry using fish that underwent *in situ* respirometry (Lab-ISMeR) and (iii) controlled laboratory respirometry on acclimated fish (Lab-Acclim). ISMeR was conducted over two consecutive years, in November 2021 and in September 2022. Controlled laboratory respirometry on individuals that underwent ISMeR or Lab-ISMeR was performed only on some individuals from September 2022 trials, after 54 days in captivity. Controlled laboratory respirometry or Lab-Acclim on acclimated individuals took place in August and September 2021 and in June 2022, for fish collected in fall 2019 and 2021, respectively (Table 1; Fig. S1).

**Table 1:** Summary of fish groups included in *in situ* (ISMeR) and laboratory (Lab-ISMeR, Lab-Acclim) respirometry experiments on redfish (*Sebastes fasciatus*)

**Group/dataset**	**Collection details**	**Experimental year**	***N* (tested fish)**	***N* (usable fish)**	**Notes**
ISMeR 2021	*In situ* trials at 25–30 m depth, HOBO probe	2021 (November)	7	5	One pump failure → 3 lost periods; survivors later died after PIT-tagging → no Lab-ISMeR follow-up
ISMeR 2022	*In situ* trials at 25–30 m depth, PyroScience probe	2022 (September)	7	5	2 periods discarded for technical malfunction, 1 fish escaped = no ISMeR-Lab comparison
Lab-ISMeR	Same fish from ISMeR 2022, tested in lab after ~54 days in captivity	2022 (November)	6	6 (4 with a paired ISMeR)	4 individuals paired with ISMeR, plus 2 Lab-only
Lab-Acclim	Collected fall 2019	2021	3	3	~2 years in captivity
Lab-Acclim	Collected fall 2021,	2022	4	4	~9 months in captivity

Collection details specify, depth, equipment and acclimation time in captivity. Experimental year indicates the testing period. *N* (tested fish) shows the number of individuals initially tested, while *N* (usable fish) indicates the number of individuals retained for analysis after excluding trials technical malfunction. Notes provide additional details on equipment failures, subsequent mortalities and relationships between groups (e.g. same individuals retested after laboratory acclimation).

#### *In situ* respirometry trials

*In situ* respirometry (ISMeR) trials were conducted in November 2021 and September 2022 using an ISMeR setup designed specifically for this study (Fig. S2). During the trials, mean bottom temperature at the deployment site was ~5°C, with natural variation between 3 and 7°C (Table 2). Two respirometers were available each year, and only the oxygen probe and their chamber housing differed between years. The assembly guide is provided in supporting information (Figure S2 and Table S1 for details on the assembly). Briefly, each setup was equipped with a 1.2-l acrylic respirometry chamber that held the fish during respirometry (30 cm length × 7.2 cm diameter; Loligo Systems, Viborg, Denmark) and a separate acrylic chamber that housed the oxygen probe. The probe chamber was a 0.77-l Loligo systems chamber (25 cm length × 6.2 cm diameter) in 2021 and a 1.2-l Loligo Systems chamber (30 cm length × 7.2 cm diameter) in 2022. Fish and probe chambers were connected to a closed loop (54 ml), made of Tygon tubing and a recirculation pump (model AD20P-1230A, Brushless DC 12V submersible pump, TCT electronics, Shenzhen, China, 4 l min^−1^) to achieve adequate water mixing throughout both chambers. A third plexiglass cylinder housed batteries and electronics. This intermittent flow respirometry setup was fixed on an 84 × 54 cm fibreglass grating using polyvinyl chloride (PVC) sheets and stainless-steel hardware to ensure that it stayed on the bottom with as little disturbance as possible (Fig. S2; Assembly guide in SI).

**Table 2:** Summary of temperature (mean ± standard deviation, median, IQR, minimum and maximum) recorded during ISMeR trials and DO saturation (mean ± standard deviation, median, minimum and maximum) obtained from the end of the flushing period during respirometry trials for each individual fish (ID) in 2021 and 2022

	Temperature					Dissolved oxygen			
ID	Mean ± SD	Median	IQR	Min	Max	Mean ± SD	Median	Min	Max
233653_2021	4.88 ± 0.46	5.12	0.25	3.72	5.30	81.43 ± 1.43	81.63	77.70	84.31
233660_2021	5.30 ± 0.21	5.46	0.34	4.92	5.68	89.65 ± 1.49	89.44	87.09	92.76
233734_2021	5.04 ± 0.23	5.25	0.42	4.42	5.38	88.15 ± 2.09	87.97	84.26	92.48
233843_2021	5.00 ± 0.26	5.02	0.26	3.96	5.50	86.36 ± 2.60	86.05	78.71	91.94
NA_2021	4.67 ± 0.52	5.04	0.40	3.64	5.24	85.12 ± 1.42	85.42	81.69	87.95
33755_2022	4.71 ± 0.74	4.31	0.62	3.31	6.02	81.30 ± 4.48	80.50	75.55	92.04
33932_2022	3.90 ± 0.71	3.87	1.17	2.61	5.94	82.22 ± 6.09	80.50	77.38	107.13
34009_2022	4.55 ± 1.04	4.04	0.75	3.20	7.10	81.44 ± 5.37	79.56	76.54	98.69
34289_2022	4.70 ± 0.61	4.83	0.40	3.48	5.81	82.40 ± 2.85	82.59	76.97	90.83
NA_2022	4.28 ± 0.99	4.02	1.26	2.99	7.10	84.35 ± 5.49	81.90	79.83	102.46

In 2021, a HOBO dissolved oxygen logger (U26-001, Onset computer Corporation, Bourne, MA, USA) measured both dissolved oxygen (DO) and temperature. DO concentration (mg l^−1^) was measured every minute. For the first two days, DO was measured using 14-min cycles consisting of a 7-min period of flush to renew the water in the respirometers followed by a 7-min closed period of which the last 6 min were used to determine oxygen uptake. Since the HOBO probe only allowed one measurement every minute, we reassessed the duration of the recirculation period, as shorter closed periods yielded fewer data points and longer periods therefore allowed more robust slope estimates. The two subsequent days, cycles lasted 20 min, with a 10-min period of flush followed by a 10-min closed period, of which the last 9 min were used to determine oxygen uptake. Considering the volume of the chamber and all parts included in the recirculation loop, we obtained a chamber to fish volume ratio between 23 and 52, depending on fish size. The oxygen probes used were calibrated with 100 and 0% (air saturation) solutions before going to the field (Table S2).

In 2022, an AquapHOx dissolved oxygen logger (PyroScience GmbH, Aachen, Germany) was used to estimate the oxygen uptake (hPa) with data being collected every second. During each trial day, DO was measured using 10-min cycles consisting of a 4-min period of flush to renew the water in the respirometers followed by a 6-min closed period of which the last 5 min were used to determine oxygen uptake. Considering the volume of the chamber and all parts included in the recirculation loop, we obtained a chamber to fish volume ratio between 24 and 66. The oxygen probes used were calibrated with 100 and 0% (air saturation) solutions before going to the field (Table S2).

Divers installed the ISMeR setups at the bottom of the collection site, fish chambers were opened, and pumps were activated to ensure that all chambers and tubes were correctly filled with water. Once the setup was stabilized and functional, two fish of appropriate size were captured with dip nets and placed as quickly as possible into the respirometry chambers. Capture and transfer were not formally timed but generally took less than 1 min per fish. Chambers were closed and oxygen uptake recording began as soon as fish were inside chambers. Oxygen uptake was monitored for ca. 24 h. Following that period, divers removed the fish from the respirometry chambers and placed them in individual cages at mid-depth relative to the capture site. This allowed gradual decompression of the swim bladder, as described above. At the end of the recording period for a given pair of fish, the ISMeR setups were brought back to the surface, data from the oxygen loggers were downloaded to a computer, batteries were changed and divers brought back the setup to the bottom and repeated the procedures with a new pair of individuals. Respirometry trials were performed on four pairs of fish each year.

In 2021, one of the recirculation pumps failed on day 2, resulting in unusable data for three out of four fish for that setup. On the fifth day, when all fish were brought up to the surface for transport, the fish used for respirometry trials were anaesthetized and identified with a PIT-tag (Biomark, Idaho, USA) associated with their ISMeR trials. Unfortunately, this additional handling caused excessive stress, and all individuals died within hours or days after arrival to the rearing facilities. Moreover, two individuals that underwent ISMeR were accidentally discarded without obtaining their mass. As no fish survived, no laboratory respirometry data were available for comparison with the *in situ* measurements of the 2021 cohort.

In 2022, one of the batteries had a connection problem, resulting in only one functioning setup being available for the second day. The issue was resolved the following day. Finally, one fish escaped from its individual cage at the end of the *in situ* trial, making it impossible to bring it back to the laboratory for subsequent lab respirometry and mass measurements.

For the two individuals with unknown mass in 2021 and the one that escaped in 2022, we assigned a body mass representing the mean for all individuals tested in the same year (Table S3).

### Lab respirometry trials (Lab-ISMeR and Lab-Acclim)

#### Lab-ISMeR

Laboratory oxygen uptake was measured for six fish brought back alive from the 2022 ISMeR deployment (Table 1: Lab-ISMeR). They were acclimated for 54 days and tested in November 2022 at a constant temperature of 6°C and a salinity of 28, which approximated the environmental conditions experienced during the field trials.

The setup included three glass chambers placed inside a rectangular tank (200 cm length × 60 cm width × 28 cm height, 336 l) with an open circulation system. Temperature was controlled by mixing cold and warm seawater. Temperature of the incoming seawater was measured with a temperature probe (Hermetically sealed RTD probe, Omega Engineering Inc., Norwalk, CT, USA) and fed to a controller (1/16 DIN Micromega autotune PID Temperature, Omega Engineering Inc.) connected to a mixing valve (sv3109, Omega Engineering Inc.) to obtain the appropriate ratio of cold and warm water (flow rate between 3.5 and 4.5 l min^−1^). The chambers were transparent but separated by opaque panels so that fish could not see each other. To achieve adequate water mixing, each chamber was connected to a recirculation loop made of Tygon tubing and a recirculation pump (1.5 l chamber: model AD20P-1230A, Brushless DC 12V submersible pump, 4 l min^−1^; 3 l chamber: model 1046, Eheim, Stuttgart, Germany, 5 l min^−1^). The volume of the recirculation loop was 16 ml for 1.5 l chambers and 52 ml for 3 l chambers. Each recirculation loop included housings for a fibre-optic oxygen probe (PyroScience Robust Oxygen Probe) as well as a temperature sensor (PyroScience P100). These were connected to a PyroScience FireSting-O2 4-channel oxygen metre combined with a PyroScience TEX4 to have temperature correction on each channel. DO levels were measured every ~ 2 s. The three chambers were also connected to a single flush pump (model 1048, Eheim, Stuttgart, Germany, 10 l min^−1^) controlled by the Aquaresp software (V3, Denmark, Aquaresp.com).

Wet mass-specific oxygen uptake (*Ṁ*O_2_, in mg O_2_ h^−1^ kg^−1^) was measured in each respirometer by intermittent-flow respirometry (Steffensen, 1989; Svendsen *et al.*, 2016) using 11.5-min cycles consisting of a 4-min period of flush to renew the water in the respirometers followed by a 7.5 min closed period to determine oxygen uptake using the slope of the decreasing DO in the respirometer during the last 6 min of the closed phase, when it had become linear. The flushing duration was sufficient to maintain DO above 95% air saturation throughout all measurement cycles.

Respirometer size was selected so as to respect a chamber to fish volume ratio between 20 and 50 (Svendsen *et al.*, 2016) and we obtained ratios between 41 and 56. The respirometry setup was cleaned before each trial with fresh water and chlorine and was rinsed thoroughly with fresh water and left to run overnight with seawater. The oxygen probes used were calibrated with 100 and 0% (air saturation) solutions before each experiment (Table S2). Fish were fasted for a minimum of 72 h prior to all respirometry experiments to ensure that they were in a post-absorptive state (Clark *et al.*, 2013; Chabot *et al.*, 2016). Each trial lasted 4 days, including preparation and execution. On day 1, the setup was cleaned, probes were calibrated, fish were selected and weighted, and then isolated for the night in a basket floating in the holding tank. On day 2, background oxygen uptake rates were estimated in each empty chamber for a minimum of 40 min before and after every respirometry trial. Each trial started with a chase protocol followed by 1 min of air exposure, a common method of estimating maximum metabolic rate (MMR) in fishes (Roche *et al.*, 2013; Rummer *et al.*, 2016) and tested on *S. fasciatus* (Guitard *et al.*, 2025, 2026). Although MMR was not considered in the present study, this procedure is reported here to provide a complete description of the experimental conditions experienced by fish prior to SMR estimation. For this, fish were transferred one at a time to a circular chase arena (65 cm height × 15 cm × diameter, 50 l, depth adjusted as a function of fish height [doubling the height of the fish in water]) using a net and chased by hand until they no longer reacted to contact and tail pinching (chase duration lasted between 70 and 131 s). The fish was then removed from the arena and held out of the water for 1 min. It was thereafter placed into a respirometry chamber, which was immediately (less than 15 s) sealed until the oxygen level reached ~80% air saturation to estimate MMR. Once the MMR estimation was completed for all individuals, control of the system was switched to regular cycles controlled by Aquaresp running the 11.5-min loops as described above, for the following ~ 70 h (days 3 and 4), with the fish left undisturbed, in the dark (except for a daily check-up with red lights), behind opaque curtains. Over this period, oxygen uptake rates of fish stabilized, and SMR could be estimated. Oxygen levels remained above 95% in the chambers during all cycles of all trials. The trials were ended at the end of day 4, when the fish were brought back to their holding tank. This protocol follows best practices for collecting and reporting respirometry data as described in Killen *et al.*, 2021 (Table S2).

#### Lab-Acclim trials

Laboratory estimates of SMR were also obtained from similarly sized *S. fasciatus* (Table S3) captured from the same site for other projects. These fish had not been used in the *in situ* study and had been maintained under laboratory conditions for up to two years before respirometry measurements were conducted (Table 1). These fish were classified into two groups according to the date of their capture and thus the length of their stay in captivity. The first group of fish was captured in Fall of 2019 and subjected to respirometry trials in August and September of 2021. The second group of fish was captured in November 2021, and their oxygen uptake was studied in June 2022 (Table 1).

Laboratory-based respirometry was performed in two experimental water baths (including the temperature control system) identical to the one described for Lab-ISMeR. These experiments involved fish of different sizes, but only seven *S. fasciatus* of similar size to those studied with ISMeR were retained here (Table S3), using the same respirometer chambers and recirculation loops used for Lab-ISMeR.

### Data extraction

For all *in situ* and laboratory trials, wet mass-specific oxygen uptake (*Ṁ*O_2_; mg O_2_ h^−1^ kg^−1^) was calculated as:


$$ {\dot{M}\mathrm{O}}_{2}=\frac{S_{{\mathrm{O}}_2}\times V}{M} $$

where ${S}_{{\mathrm{O}}_2}$ is the slope of oxygen concentration against time during the closed measurement phase (mg O_2_ l^−1^ h^−1^), *V* is the effective water volume of the respirometry system (l), and *M* is fish wet mass (kg). Slopes initially obtained from oxygen partial pressure (*P*O_2_; kPa h^−1^) were converted to oxygen concentration slopes using oxygen solubility at the corresponding water temperature and salinity (Garcia and Gordon, 1992). Effective water volume included the respirometer and its recirculation loop and was corrected for the volume displaced by the fish. Fish mass was used to estimate fish volume assuming a density of 1 g ml^−1^. The same calculation was used for both *in situ* and lab measurements, with differences in background-respiration correction described below.

#### *In situ* data extraction

For each sensor type, custom R scripts were developed to extract, clean and structure the data. Raw files were batch-processed using metadata entries that included the time of fish placement into and removal from the respirometer, as well as the durations of flush, wait and measurement phases for each trial.

To determine the timepoints corresponding to oxygen uptake measurements, an automated routine first identified a reliable anchor point (a clear end of a measurement slope), from which previous and subsequent cycles were generated based on the known cycle duration. All extracted cycles were visually inspected using automatically generated plots to verify the positioning of slope boundaries. This step was more challenging for the 2021 HOBO data because fewer data points were available during each measurement period as the probe recorded DO only once per minute rather than every two seconds in 2022 with the PyroScience system. During this step, two fish from the 2022 trials were rejected because there were apparent signs of a malfunctioning system likely due to air remaining in the setup when the fish was introduced into the respirometer.

To obtain *Ṁ*O_2_ for each fish, only the periods corresponding to the closed measurement phases were retained. In 2021, the HOBO logger recorded DO concentration (mg l^−1^) without an onboard salinity correction (salinity setting = 0). Mean salinity during the trials, measured independently using a HOBO U24-Conductivity logger, was 30 ppt. DO concentration was converted to oxygen partial pressure (*P*O_2_, kPa) using this salinity value and the corresponding temperature. In 2022, the AquapHOx logger recorded *P*O_2_ directly in hPa.

Oxygen uptake rates were computed using the fishMO2 package (Chabot *et al.*, 2016; Chabot, 2020). The calcSMR function of fishMO2 was used to calculate a low and repeatable level of metabolic rates in the respirometers (quantile *P* = 0.2) of the *Ṁ*O_2_ values obtained for a period of ca. 24 h of data after 3 h acclimation. We applied quantile *P* = 0.2 to obtain a standardized estimate of the lower *Ṁ*O_2_ distribution using the same extraction method as for the laboratory data in this study as well as others on the same species under comparable conditions (Guitard *et al.*, 2025, 2026). However, because the field fish were not necessarily post-absorptive and their activity was not quantified, fully settled measurements may have been infrequent and q0.2 may therefore include some activity-related metabolism. Accordingly, we interpret this estimate as RMR, rather than SMR. For *in situ* trials, background respiration was not measured or subtracted because additional measurements would have reduced the number of fish studied during the 5 days field trips. We expected it to be negligible due to the short duration of the trials and the low water temperatures. This assumption is supported by independent laboratory measurements under similar conditions, where microbial respiration accounted for only a minimal fraction of total *Ṁ*O_2_ (Guitard *et al.*, 2025, 2026). Low *R*^2^ slopes in the *in situ* trials were typically associated with unsettled fish or air intrusions. To avoid excluding valid low *Ṁ*O_2_ values, each decline was visually inspected, and the minimum *R*^2^ was chosen (between 0.7 and 0.95) using the diagnostic approach of (Chabot *et al.*, 2020), selecting a minimum *R*^2^ that rejected non-linear declines in DO but retained linear ones. Summary statistics of DO and temperature at the end of the flush period were used to estimate ambient sea water oxygenation and temperature (Table 2).

#### Laboratory data extraction

Wet mass-specific oxygen uptake rates (*Ṁ*O_2_; mg O_2_ h^−1^ kg^−1^) were calculated from the slopes of linear regressions between DO (kPa) and time. Background respiration was estimated from empty respirometers before and after the fish trial and subtracted assuming a linear increase from start to end of the experiment. Using the diagnostic evaluation of *R*^2^ values recommended by (Chabot *et al.*, 2020), measurement slopes were visually inspected across a range of *R*^2^ values. An *R*^2^ threshold of 0.85 was selected as the lowest value that consistently retained visually linear declines in oxygen while excluding slopes showing clear deviations from linearity. All slopes exceeded this threshold for every individual except one, which had a rejection rate of 2.84%.

SMR was estimated with the fishMO2 package (Chabot *et al.*, 2016; Chabot, 2020) as the quantile (*P* = 0.2) of the *Ṁ*O_2_ values obtained for a period of ca. 72 h after an acclimatation period of 10 h. Because we have observed night activity in this species, we have estimated SMR for each individual with and without nights, retaining the lower estimate as our best estimate of SMR.

### Statistical analysis

#### Comparison of RMR for ISMeR between both years

To assess the assumptions for the comparison of RMR from ISMeR between years, we used Shapiro–Wilk tests, which detected no significant departure from normality (Shapiro–Wilk test, *P* > 0.3 for both years), and Levene’s test, which provided no significant evidence of unequal variance between years (*P* = 0.08). Although Levene’s test provided no significant evidence of unequal variances, we used Welch’s *t*-test to compare RMR between years because it does not assume equal variances, which are difficult to assess reliably with small samples.

#### Comparison of SMR for Lab-ISMeR and Lab-Acclim fish

To evaluate whether SMR differed between fish measured in the laboratory after *in situ* respirometry (Lab-ISMeR) and laboratory fish that had never undergone *in situ* respirometry (Lab-Acclim), we used Welch’s *t*-test. The two groups comprised distinct PIT-tagged individuals, and each fish contributed a single laboratory SMR estimate; therefore, observations in this comparison were independent. This test was chosen because sample sizes were small and unequal (Lab-ISMeR: *n* = 6; Lab-Acclim: *n* = 7) and it does not require equal group variances as for the comparison between years.

#### Comparison of RMR from ISMeR to SMR in the lab

To extend comparisons beyond individuals measured in both settings, we compared metabolic rates between fish measured *in situ* (ISMeR) and those measured in the laboratory (Lab). The ISMeR group comprised four individuals from 2021 (unpaired) and four individuals from 2022, which were also tested in the laboratory and paired for this analysis. The Lab group comprised six Lab-ISMeR fish (four paired, see above, and two individuals measured successfully in the lab but with no valid RMR obtained *in situ* because of technical issues) and seven Lab-Acclim fish. Because the dataset contained both paired observations (2022 ISMeR–Lab-ISMeR) and independent observations (ISMeR 2021, some Lab-ISMeR and Lab-Acclim), we applied the partially overlapping samples *t*-test using the R package ‘Partiallyoverlapping’ (Derrick *et al.*, 2017a, 2017b). This test combines information from paired and unpaired data without discarding observations and interpolates between the paired *t*-test and Welch’s test depending on the data structure.

#### Comparison of RMR from ISMeR and SMR from Lab-ISMeR

To compare metabolic rates measured *in situ* (ISMeR) and in the laboratory (Lab-ISMeR) for the same individuals sampled in 2022, we used a paired *t*-test. This test evaluates whether the mean within-individual difference between ISMeR and Lab measurements differs from zero.

Given the small sample size (*n* = 4 pairs), assumptions of equal variance could not be reliably evaluated; thus, Welch’s adjustment for unequal variance was applied. Body mass was also compared between the two experiments using the same paired test structure.

## Results

### Abiotic factors variability

Median temperatures recorded during ISMeR trials ranged from 3.87 to 5.46°C, and temperature remained close to 5°C during most of the time, for most of the trials. Mean temperatures were similar, varying from 3.90 to 5.30°C, and the standard deviations remained low (0.21–1.04°C), reflecting limited thermal fluctuation during individual trials. These results indicate that, despite minor inter-individual differences, the thermal environment was generally stable during measurements, which is reflected by the interquartile range (IQR) for most individuals (Fig. 2; Table 2). Most fish experienced steady conditions throughout the measurements, with values staying close to the average and showing little variation over time. However, the thermal range in 2022 was broader than in 2021, with maximum values reaching 7.10°C for some individuals and generally higher IQRs. Short increases in *Ṁ*O_2_ occasionally coincided with small temperature peaks, particularly in 2022 (Fig. S4). Despite these within-trial patterns, RMR did not differ significantly between years (Fig. 3A).

**Figure 3:**
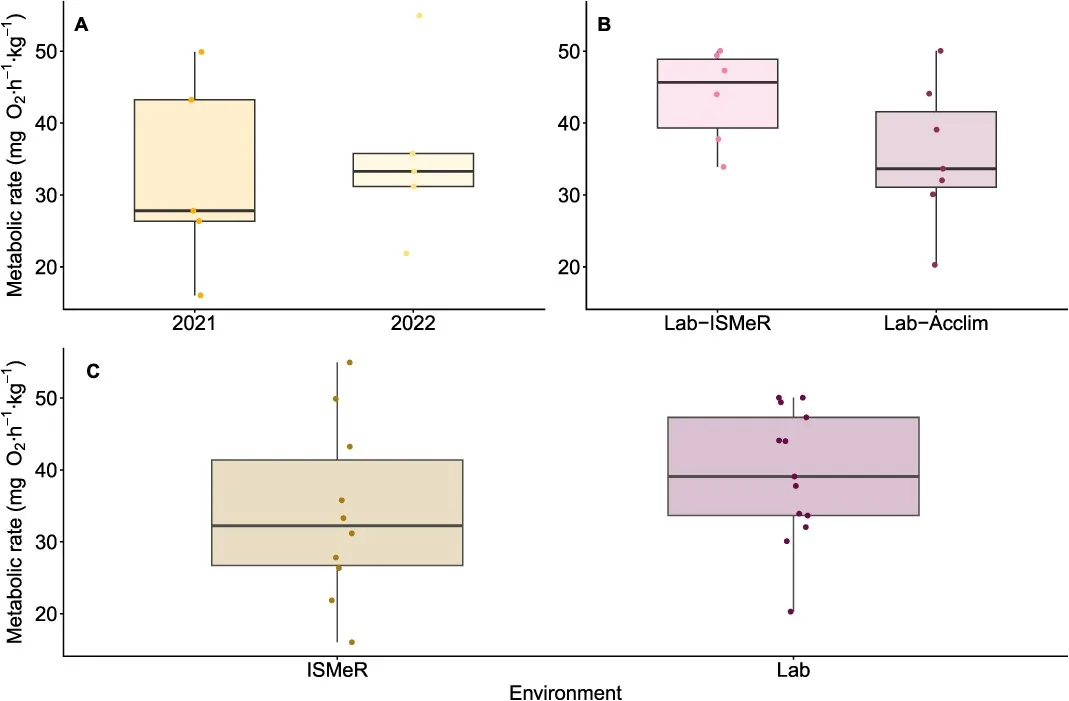
*Ṁ*O_2_ (mg O_2_ h^−1^ kg^−1^) of redfish (*Sebastes fasciatus*). (A) ISMeR in 2021 using the HOBO probe (*n* = 5) vs. 2022 using the PyroScience probe (*n* = 5). (B) Laboratory measurements for fish previously tested *in situ* (Lab-ISMeR; *n* = 6) and laboratory-acclimated fish (Lab-Acclim; *n* = 7). (C) *Ṁ*O_2_ across environments (ISMeR vs. Lab), pooling years within ISMeR and pooling Lab-ISMeR with Lab-Acclim within Lab. In all panels, boxes span the interquartile range (IQR), horizontal lines mark the median, and whiskers extend to the most extreme observations within 1.5 × IQR of the box edges. Each point represents an individual fish’s measurement. For statistical analysis, data were grouped by environment (ISMeR vs. Lab), with both paired and unpaired observations accounted for using a partially overlapping samples *t*-test.

For DO, medians ranged from 79.56 to 89.44% saturation, and mean DO saturation ranged between 81.30 and 89.65% saturation. Standard deviations were generally low (1.42–6.09% saturation), indicating that most fish experienced steady oxygen conditions during the respirometry trials, at levels known not to affect SMR (Guitard *et al.*, 2026). Nevertheless, a few exceptions were observed in 2022, with SDs of ambient DO reaching up to 6.09% saturation (Table 2).

### Comparison of metabolic rates among years, fish groups and measurement settings

No statistically significant differences were detected in any of the comparisons of metabolic rates among years, fish groups, or measurement settings (Figs 3 and 4). Among the ten fish successfully tested *in situ*, five were measured with the HOBO probe in 2021 and five with the PyroScience probe in 2022. Greater inter-individual variability was observed in 2021 than in 2022 (Fig. 3A). However, RMR did not differ significantly between fish measured in 2021 (mean = 32.65 mg O_2_ h^−1^ kg^−1^) and those measured in 2022 (mean = 34.40 mg O_2_ h^−1^ kg^−1^; Welch’s *t*-test: *t* = −0.33, df = 8, *P* = 0.75; Fig. 3A).

**Figure 4:**
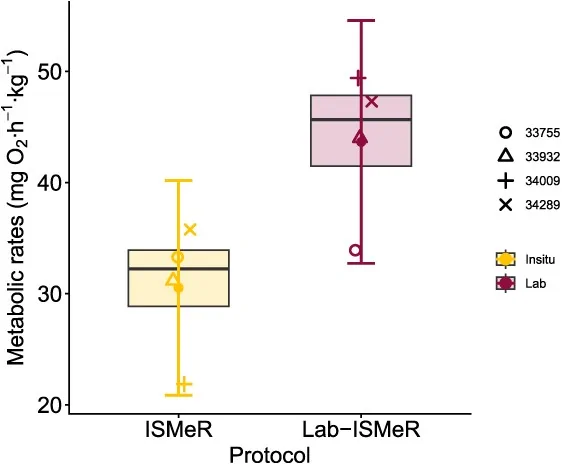
*Ṁ*O_2_ (mg O_2_ h^−1^ kg^−1^) for redfish (*Sebastes fasciatus*) that were measured during *in situ* respirometry (ISMeR) and on the same fish in the laboratory (Lab-ISMeR) *n* = 4. Boxplots show medians, IQRs, and 1.5 × IQR whiskers; overlaid symbols give group means ±95% CI. Point shape denotes PIT tag to visualize repeated measures.

Similarly, laboratory SMR did not differ significantly between Lab-ISMeR fish (*n* = 6, mean = 43.7 mg O_2_ h^−1^ kg^−1^) and Lab-Acclim fish (*n* = 7, mean = 35.6 mg O_2_ h^−1^ kg^−1^; Welch’s *t*-test: *t* = −1.78, df = 10.5, *P* = 0.10; Fig. 3B).

When all available measurements were considered, metabolic rate did not differ significantly between RMR measured *in situ* and SMR measured in the laboratory (partially overlapping Welch *t*-test: *t* = −1.27, df = 10.4, *P* = 0.23; Fig. 3C). This comparison included 10 ISMeR fish—5 measured in 2021 and 5 in 2022—and 13 laboratory fish, comprising 6 Lab-ISMeR fish and 7 Lab-Acclim fish. Four of the Lab-ISMeR fish had paired *in situ* and laboratory measurements.

For these four individuals, mean RMR measured *in situ* was 30.52 ± 6.07 mg O_2_ h^−1^ kg^−1^ (mean body mass: 58.83 ± 20.55 g), whereas mean SMR measured in the laboratory was 43.65 ± 6.86 mg O_2_ h^−1^ kg^−1^ (mean body mass: 61.38 ± 20.24 g). The difference was not statistically significant (paired *t*-test: *t* = −2.15, df = 3, *P* = 0.12; Fig. 4). Body mass also did not differ significantly between the *in situ* and laboratory measurements of these individuals (mean difference = 2.6 g; paired *t*-test: *t* = −0.32, df = 3, *P* = 0.77).

## Discussion

To the best of our knowledge, this is the first study to measure *Ṁ*O_2_ in *S. fasciatus in situ*, offering a rare alternative to laboratory-based approaches that are often limited by the challenges of capturing and transporting this physoclistous species alive and in good condition. This is also the first study in fish to compare *in situ* and laboratory-based *Ṁ*O_2_ measurements on the same individuals, hinting on how experimental conditions may influence aerobic metabolic performance relative to the natural environment.

We initially predicted that metabolic rates measured *in situ* would exceed lab-based SMR values due to uncontrolled abiotic (temperature, oxygenation and salinity) and biotic variables (digestion, spontaneous activity and environmental stimuli) and to the relatively short duration of field trials, which means that the fish may not have had time to acclimate to the respirometer and display *Ṁ*O_2_ values typical of SMR. However, no significant differences were detected between the best possible estimation of RMR obtained *in situ* and SMR in the lab on some of the same individuals and other similar size fish acclimated to laboratory conditions. This suggests that metabolic data collected *in situ* can provide estimates of maintenance costs for *S. fasciatus* in the field that are comparable to those obtained under laboratory conditions. Nonetheless, the limited sample size and the inclusion of three individuals with estimated mass (two in 2021 and one in 2022; not part of the same-individual comparison) highlight the need for further replication to confirm these findings. Despite these limitations, the consistency observed on repeated individuals supports the potential of ISMeR as a valuable approach to estimate the rate of energy expenditures under relevant environmental conditions, and this approach is particularly useful for species that are difficult to transport or maintain in captivity. ISMeR offers clear benefits and applications in conservation physiology and the study of sensitive taxa.

The absence of statistical differences between field and lab estimates of maintenance metabolic rates should, however, be interpreted cautiously, as the small sample size for the ISMeR vs. Lab-ISMeR comparison strongly limited statistical power. Contrary to our initial hypothesis, *in situ* RMR showed a trend for lower values than laboratory-derived SMR. This result suggests that a larger number of observations or longer *in situ* trials could bring the current trend to statistical significance. Indeed, in our previous work on *S. fasciatus* (Guitard *et al.*, 2025, 2026), a stable SMR was rarely reached within the first 24 h of measurement. In Guitard *et al.* (2025, 2026), more reliable estimates of SMR were obtained after 48 h, as *Ṁ*O_2_ gradually declined and stabilized under controlled laboratory conditions. These long periods with elevated *Ṁ*O_2_ were likely partly due to recover from the fish being chased to elicit MMR just before placement in the respirometer. A similar period was required before low and stable values of *Ṁ*O_2_ were observed in some of the *in situ* trials as well. Although fish were carefully and rapidly placed in the respirometers to minimize handling stress, we could not determine whether these variable rates were caused by handling, confinement, or digestion. Nevertheless, it is likely that extended *in situ* trials would reveal further declines in *Ṁ*O_2_, resulting in lower estimates of RMR.

Several biological factors may also have contributed to variation in *Ṁ*O_2_ during the trials. In addition to the gradual decline observed after capture and handling, short increases in *Ṁ*O_2_ occurred at different times among individuals and may have been caused by spontaneous activity within the chambers. Some of these changes may also have been related to diel activity or changes in ambient light. In the laboratory, we have observed increased activity during the night in this species, whereas fish measured *in situ* remained exposed to natural changes in light and other environmental cues. Although we did not identify a consistent change in *Ṁ*O_2_ around dawn or dusk during the *in situ* trials, the short trial duration and limited sample size prevented us from ruling out an effect of light transitions or formally assessing diel patterns. *Sebastes fasciatus* can also be observed in aggregations, and confinement of individuals may have altered their normal interactions with other fish. However, we could not determine whether the absence of these interactions influenced their activity or metabolic rate. Although territorial behaviour was not formally evaluated, we have not observed obvious territorial or aggressive behaviour during six years of maintaining this species in captivity.

As noted in our predictions, capturing fish directly from their natural habitat prevented us from assessing their digestive state, which may have influenced the *Ṁ*O_2_ measured during the early phase of the trials. Longer ISMeR trials would help reduce this uncertainty by allowing time for digestion to be completed. Another possible approach would be to control food intake prior to respirometry, for example by equipping respirometers with bait to lure fish inside and automatic closing doors, as suggested by Drazen and Yeh (2012). This technique would reduce handling because fish would enter the chambers on their own, but it introduces new challenges, fish may not consume all the bait, and uneaten food could increase bacterial respiration. Even with such methods, extended *in situ* trial durations would remain essential to obtain reliable RMR estimates.

An additional consideration is the abiotic environment in which our trials were conducted. At Les Escoumins in the St. Lawrence Estuary, bottom temperature (~30 m: 5°C on average) and DO (80–90% saturation) remained relatively stable during the *in situ* measurements despite the possible effect of tide-induced movements of water masses. At these depths, fish likely remained below the seasonal thermocline, where temperatures are consistently cold and less affected by rapid surface fluctuations. In early fall, although surface waters start to cool, waters at ~30 m remain stable between 2 and 7°C, a pattern maintained by seasonal stratification and the persistent cold intermediate layer (Galbraith *et al.*, 2025). Although less variable than surface waters, temperatures at ~30 m still fluctuate seasonally, typically ranging from near 0°C in winter to around 6–7°C in late summer and early fall, depending on local mixing and stratification (Galbraith *et al.*, 2025). In addition to this seasonal pattern, the area is known for tidally driven upwelling events caused by strong currents and complex bottom topography, which can bring deeper, colder water towards mid-depths (Cyr *et al.*, 2015; Bluteau *et al.*, 2021). These events may cause occasional short-term drops in temperature, even in warmer months. However, because currents and water-mass movements were not monitored, we cannot determine whether the observed fluctuations were caused by upwelling. Overall, the thermal environment was consistent and representative of the cold, near-bottom conditions typically encountered by *S. fasciatus* in late fall at Les Escoumins, providing stable yet realistic conditions for metabolic measurements during that period.

In laboratory settings, fish metabolic rates usually increase with temperature across the species’ natural range. In contrast, natural environments are rarely thermally constant. Work on Atlantic salmon (*S. salar*) has shown that diel temperature fluctuations can influence SMR depending on acclimation temperature and population origin (Oligny-Hébert *et al.*, 2015), and more broadly, that environmental variability can substantially shape metabolic responses in ways that differ from stable laboratory settings (Enders and Boisclair, 2016). In our trials, mean bottom temperature was near 5°C, but natural variations between 3 and 7°C occurred during *in situ* trials. *In situ* measurements occasionally showed short increases in *Ṁ*O_2_ that coincided with small temperature peaks (Fig. S4). When examining each year separately, the relationship between RMR and temperature showed no significant variation in 2021, whereas a positive relationship was detected within the temperature range experienced by fish in 2022 (Fig. S4). However, this within-year effect was small and did not translate into differences in overall RMR between years: mean RMR did not differ significantly between all fish tested in 2021 and all fish tested in 2022 (Fig. 3A). Body mass was also unlikely to explain the variability between years, as mean mass was similar in 2021 and 2022 (53.85 and 58.23 g, respectively). Although the range was broader in 2022 (31.42–80.54 g) than in 2021 (32.10–67.97 g; Table S3), there was no clear systematic difference in body size between years. Fish captured in 2021 and 2022 may nevertheless have experienced different temperature conditions before capture. Such differences in thermal history could have influenced their short-term metabolic responses during respirometry and contributed to the greater inter-individual variability observed in 2021. However, because the thermal histories of individual fish before capture were unknown, their contribution to interannual variation could not be quantified. This indicates that the quantile-based approach effectively captures resting metabolic rates despite minor within-trial temperature variation, and that *S. fasciatus* maintained stable maintenance costs under the relatively constant thermal conditions encountered *in situ*. Thus, while *in situ* conditions at the study site facilitated comparison with laboratory experiments under stable conditions, this type of comparison may be more complex if *in situ* conditions were more variable, possibly necessitating comparison with laboratory experiments under variable abiotic conditions. Caution is warranted when extending these findings to species or habitats with greater environmental variability. For example, in freshwater environments or shallow marine habitats, temperature and oxygen can fluctuate rapidly due to diel cycles, river inflows or seasonal changes. Such variability can complicate the interpretation of ISMeR data, but it also provides valuable insight into how fish cope with unstable environmental conditions.

## Future Improvement of the Setup

Few studies have successfully measured the metabolic rate of fishes *in situ* (Farrell *et al.*, 2003; Drazen and Seibel, 2007; Drazen and Yeh, 2012), and our work represents a rare attempt to do so. However, within the context of high complexity and cost of such experiment, several technical challenges resulted in a reduced number of observations relative to the initial plan. In particular, the setup built sometimes required on-site repairs, and some valuable data were likely lost to the retention of air in the circuit. To minimize the occurrence of such issues in the future, the use of sturdier pumps would provide more consistent water flow to effectively flush out any residual air and thus decrease the risk that air remains trapped in the chambers or tubing, while helping prevent damage from debris. Visual inspection to confirm the absence of air when the setup is brought under the surface would also help in preventing this problem.

Another important source of variability was the choice of oxygen probe. In the present experiment, we tested two probes: a HOBO probe characterized by a minimum recording frequency of one measurement per minute, and a PyroScience probe, which provided recordings every second. This comparison highlighted the trade-off between affordability and data quality. While metabolic rates could be extracted from both probes, the PyroScience system generated more robust and reliable data. We did not see a statistical difference between the two probes (as shown by the 2021 vs. 2022 comparison; Fig. 3A), but the PyroScience data (2022) showed less variation overall. Also, the higher acquisition frequency of the PyroScience probe made data extraction easier and more consistent by providing a greater number of datapoints for calculating reliable slopes. Since the completion of this experiment, newer probes have become available from PyroScience and other manufacturers. Based on our experience, we strongly recommend investing in high-frequency, high-precision sensors, as the quality of the oxygen signal is fundamental to the success of ISMeR.

In conclusion, our study provides the first comparison between *in situ* and lab-based aerobic metabolic rates in the same fish. This comparison showed that this new ISMeR setup yields estimates similar in magnitude to laboratory-based SMR. These findings support the use of this setup for generating ecologically relevant metabolic data from fish in general, but in particular for physoclistous species that are difficult to study using traditional laboratory respirometry. For *S. fasciatus*, field-based estimates of maintenance metabolism could improve our understanding of physiological vulnerability and habitat suitability as the Gulf of St. Lawrence continues to warm and lose oxygen. Future studies should focus on increasing sample size, extending the duration of *in situ* trials, and controlling for digestive state in order to better capture baseline metabolic demand. These improvements will help refine ISMeR as a conservation-oriented tool for studying the physiology of hard-to-access aquatic species.

## Supplementary Material

Web_Material_coag090

## Data Availability

Data generated or analysed during this study are available in the Université du Québec à Rimouski’s Dataverse Collection within the Borealis Repository, at https://doi.org/10.5683/SP4/RAOPY3.
