# Supplementary material for: Bridging lab and field: in situ oxygen uptake measurements match laboratory-derived values in Sebastes fasciatus
Source: Conserv Physiol. 2026 Nov 12;14(1):coag090. doi: 10.1093/conphys/coag090 (PMC13759192; doi:10.1093/conphys/coag090)
Supplement: Supplementary file 1 [file web_material_coag090.zip › Supplemental_information__In_situ (1).docx]

Bridging lab and field: *In situ* oxygen uptake measurements match laboratory-derived values in *Sebastes fasciatus*

Joëlle J. Guitard^1*^, Denis Chabot^2^, Dominique Robert^1^, David Deslauriers^1^

^1^ Institut des sciences de la mer, Université du Québec à Rimouski, Rimouski, QC, Canada

^2^ Maurice Lamontagne Institute, Fisheries and Oceans Canada, Mont-Joli, QC, Canada

*Corresponding author: Joëlle J. Guitard; joelle.guitardbio@gmail.com

# Supplemental information

Figure S1. Flow diagram of fish tested and retained for *in situ* and laboratory respirometry in *Sebastes fasciatus*. Fourteen fish underwent *in situ* trials in 2021 and 2022, yielding ten valid observations (ISMeR; five per year). Four fish also provided laboratory measurements for the paired comparison. Laboratory data comprised six fish previously tested *in situ* (Lab–ISMeR; four paired and two with laboratory data only) and seven laboratory-acclimated fish (Lab–Acclim). Exclusions and reasons for the absence of laboratory follow-up are indicated in the manuscript.

ASSEMBLY GUIDE RESPIROMETER V1.0

Author: Frédérik Bélanger

Project: Respirometer V1.0

This assembly guide presents the general steps to assemble the respirometer V1.0. It does not include all dimensions and steps to fully build the system but is directed to explaining the general steps and resources to assemble the respirometer. To find more information about the different parts used for this system, refer to the parts lists and the manufacturer’s specification sheets.

First, the underwater housing from Blue Robotics was assembled following the manufacturer’s instruction on their website. This housing has a custom acrylic tube length to accommodate for the batteries used to power the system. It includes an electronic tray to mount all the electronics. The underwater bulkhead connector, the underwater switch, the two blank bulkheads, and the vent bulkhead were mounted on the endcap of the housing. The bulkhead connector was installed by taping the hole on the endcap to the proper thread size. The pumps were connected to the in-line underwater connector using an underwater splicing kit from 3M and following the pin out presented on Table 1.

The electronics were then mounted in the housing. This includes the Arduino board, the relay mountable board, the 12V-5V power supply, the potentiometer, the Subcon connectors, the underwater switch, the batteries, the real-time clock, and the LCD screen. The electronics were connected following the following schematics (Figure 1). The Arduino is powered by the 12V to 5V converter from Blue Robotics and the power is controlled by the underwater switch. The LCD screen and real-time clock are powered from the Arduino 3.3V and communicate using the I2C (SDA and SCL) pins in parallel. The LCD screen is used to display the start time of the cycles and the cycle time. The potentiometer is connected to 3.3V and ground of the Arduino. The cursor pin is then connected to analog pin 0 of the Arduino. The voltage measure on pin 0 is used to control the cycle times before it starts. The pumps are powered directly by the battery and controlled by the relays on the Arduino. The bulkhead connector pin out is presented in Table 1. The charging pins are meant to connect another in-line connector to the bulkhead so that the batteries can be charged without opening the housing. A fuse holder and fuse were added on the VCC+ lead from the batteries.

More information about the programming of the Arduino can be found in the file itself.

Once the electronics were completed, a clamp system was made using the threaded rods, stainless steel hardware and PVC sheets to hold the housing shut since there is no safety on the housing and it relies only on outside pressure to stay closed. In the next version of the housings from blue robotics, this is not necessary since the housing comes with a retaining nylon ring.

The 2 Loligo chambers, the underwater housing, the pumps, and the lifting hardware were installed on the fibreglass grating using PVC sheets and stainless-steel hardware. Pump 2 was then connected using Tygon tubing to the organism chamber and one check valve so that it would flush the water from the chambers. A check valve was added to the output of the organism chamber so that the water could not go back inside. Pump 1 was mounted in between the two chambers so that the sensor chamber would get water from the organism chamber. A tube was then installed in between the two chambers to complete the loop. Plastic knobs were added to the organism chamber to make it easier for divers to open the chamber. Those knobs were attached using a small wire to make sure they won’t get lost underwater. All the tubing was secured using hose clamps. The full assemble of the respirometer is shown in Figure 2.

Figure S2: Electronics schematics

Table S1: Underwater connectors pin out

| Inside housing connections | Bulkhead and in-line pins | Outside housing connections |
| --- | --- | --- |
| Relay 1 VCC | 1 (black) | Pump 1 (sensor) VCC |
| GND | 2 (white) | Pump 1 and 2 GND |
| Relay 2 VCC | 3 (red) | Pump 2 (flush) VCC |
| GND | 4 (green) | GND charging pin |
| Battery VCC | 5 (orange) | VCC charging pin |
| Battery VCC | 6 (blue) | VCC charging pin |

*Figure S3: Assembled system*

Table S3. Metadata for redfish individuals used across all respirometry trials. Shown are the experiment type (in situ ISMeR using HOBO or Pyro probes, and laboratory-acclimated respirometry), individual identification (PIT-tag), wet mass at the time of testing, year of collection, and year of measurement. Grey-highlighted masses correspond to individuals for which body mass was not recorded and subsequently assigned as the mean mass of all other individuals tested in the same year.

| Experiment | ID | Mass (g) | Collection year | Testing year |
| --- | --- | --- | --- | --- |
| ISMeR - HOBO | 233653 | 54.35 | 2021 | 2021 |
| ISMeR - HOBO | NA | 54.35 | 2021 | 2021 |
| ISMeR - HOBO | 233843 | 60.5 | 2021 | 2021 |
| ISMeR - HOBO | 233734 | 67.97 | 2021 | 2021 |
| ISMeR - HOBO | 233660 | 32.1 | 2021 | 2021 |
| ISMeR - Pyro | 34009 | 31.42 | 2022 | 2022 |
| ISMeR - Pyro | NA | 55.81 | 2022 | 2022 |
| ISMeR - Pyro | 33932 | 57.91 | 2022 | 2022 |
| ISMeR - Pyro | 33755 | 80.54 | 2022 | 2022 |
| ISMeR - Pyro | 34289 | 65.46 | 2022 | 2022 |
| Lab-ISMeR | 34009 | 34.65 | 2022 | 2022 |
| Lab-ISMeR | 34289 | 66.14 | 2022 | 2022 |
| Lab-ISMeR | 33871 | 72.5 | 2022 | 2022 |
| Lab-ISMeR | 34088 | 33.26 | 2022 | 2022 |
| Lab-ISMeR | 33932 | 61.18 | 2022 | 2022 |
| Lab-ISMeR | 33755 | 83.56 | 2022 | 2022 |
| Lab-Acclim | 234092 | 95.55 | 2019 | 2021 |
| Lab-Acclim | 233922 | 72.96 | 2019 | 2021 |
| Lab-Acclim | 234126 | 71.74 | 2019 | 2021 |
| Lab-Acclim | 33721 | 57.18 | 2021 | 2022 |
| Lab-Acclim | 33640 | 72.57 | 2021 | 2022 |
| Lab-Acclim | 33738 | 32.2 | 2021 | 2022 |
| Lab-Acclim | 33599 | 82.44 | 2021 | 2022 |

Figure S4*.* Relationship between individual mass-specific oxygen consumption rate (ṀO₂, mg O₂ h⁻¹ kg⁻¹) and water temperature (°C) for all non-excluded *in situ* respirometry trials on redfish (*Sebastes fasciatus*). Panels are grouped by year (2021 first, then 2022); each panel shows one fish (session–system and PIT label). The x-axis is adaptive per panel; the y-axis is fixed within each year to aid visual comparison across individuals. A linear model (ṀO₂ ~ temperature) is fit per panel (solid line); the printed *p*-value is the two-sided t-test for the slope (H₀: β₁ = 0), and *R*² is the coefficient of determination from that model.
