# Supplementary material for: Bridging lab and field: in situ oxygen uptake measurements match laboratory-derived values in Sebastes fasciatus
Source: Conserv Physiol. 2026 Nov 12;14(1):coag090. doi: 10.1093/conphys/coag090 (PMC13759192; doi:10.1093/conphys/coag090)
Supplement: Supplementary file 1 [file web_material_coag090.zip › TableS2.pdf]

TABLE S2A. Checklist of 53 essential criteria for the reporting of methods for aquatic intermittent-flow respirometry for the *in situ* respirometry (ISMeR) and the laboratory respirometry.

Number	Criterion	Response In situ	Response Lab	Value In situ	Value Lab
1	Body mass of animals at time of respirometry	Yes	Yes	Mean assigned for unknown masses; size-appropriate individuals used; Table S3	Table S2
2	Volume of empty respirometers	Yes	Yes	1.2 L (fish chamber); probe chamber 0.77 L (2021) or 1.2 L (2022)	1.5 and 3.0 L
3	How chamber mixing was achieved	Yes	Yes	Closed loop with Tygon tubing + 12V submersible pump	Tygon tubing + recirculation pump
4	Ratio of net respirometer volume to animal body mass	Yes	Yes	23–52 (2021); 24–66 (2022)	41-56
5	Material of tubing used in any mixing circuit	Yes	Yes	Tygon	tygon
6	Volume of tubing in any mixing circuit	Yes	Yes	54 mL closed loop	16 mL (for the 1.5L)<br>53mL (for the 3.0L)
7	Tubing volume included in calculations?	Yes	Yes	Included in chamber-to-fish ratio	Yes
8	Material of respirometer	Yes	Yes	Acrylic	glass

9	Type of oxygen probe and data recording	Yes	Yes	2021: HOBO U26-001; 2022: AquapHOx (PyroScience)	PyroScience Robust probe; FireSting-O2 + TEX4
10	Sampling frequency of water dissolved oxygen	Yes	Yes	2021: 1/min; 2022: 1/s	~ every 2seconds
11	Placement of oxygen probe	Yes	Yes	In dedicated probe chamber connected to loop	In recirculation loop
12	Flow rate during flushing and recirculation	Yes	Yes	Recirc pump 4 L min^{-1}; flush durations described but no flow rate given	4–5 L/min recirc; 10 L/min flush
13	Timing of flush/closed cycles	Yes	Yes	2021: 7/7 min then 10/10 min; 2022: 4/6 min	4-min flush; 7.5-min closed
14	Wait time excluded from closed measurement cycles	Yes	Yes	Last 6 min (2021 first cycles), last 9 min (2021 long cycles), last 5 min (2022)	First 1.5 min excluded; slope over last 6 min
15	Probe calibration	Yes	Yes	Sodium sulphite was used to obtain a 0% solution for calibration. Was done once before going to the field, so once before four days of use.	Before each experiment (0% & 100%)<br>Sodium sulphite was used to obtain a 0% solution for calibration.
16	Software temperature compensation	2022 only	Yes	AquapHOx has integrated temp compensation (implicit)	Via TEX4
17	Temperature during respirometry	Yes	Yes	Natural variability measured by both probes	6°C
18	How temperature was controlled	Yes	Yes	Ambient seawater; Natural variability	RTD probe + PID controller + mixing valve

19	Photoperiod during respirometry	Yes	Yes	Natural darkness at depth	Dark; red-light daily check
20	Ambient water bath cleaning/aeration	Not applicable	Yes	In situ seawater environment	Freshwater+chlorine; rinse; overnight seawater
21	Total volume of ambient water bath	Not applicable	Yes	Open ocean environment	336L
22	Minimum dissolved oxygen during closed phases	Yes	Yes	Table 1; determined from probe readings	~80% for MMR, >95% for SMR
23	Chambers visually shielded?	No	Yes	Animals had access to the open water	
24	Animals per trial	Yes	Yes	1 fish per setup, 2 setup per trial	3
25	Animals able to see each other?	yes	Yes	They could see all other organisms around them, but not the other respirometry setup	Not able
26	Duration of fasting before placement	Not stated	Yes	Impossible to know the state of the fish	≥72hours
27	Duration of all trials combined	Yes	Yes	~24 h per pair; 4 pairs per week, one week per year	4 days per trial
28	Acclimation time before respirometry	Not applicable	Yes	Wild fish; immediate measurement after capture	54 days
29	Background respiration measured?	No	Yes		Empty chamber before and after
30	Duration/number of background slopes	No	Yes		≥40 min before and after

31	Model for background respiration	No	Yes		Linear
32	Background respiration level	No	Yes		
33	System cleaning	Not applicable	Yes	In situ deployment	Freshwater + chlorine; overnight seawater between each trials
34	Acclimation time after transfer	Yes	Yes	3 hours before using data for RMR	
35	Time period over which oxygen uptake was measured	Yes	Yes	~24 hours	>24 h
36	Value taken as SMR/RMR	Yes	Yes	RMR derived from closed-cycle slopes with 0.2 quantile	0.2 quantile, Chabot et al., 2016
37	Number of slopes used	Not stated	Yes		
38	Time periods removed	Yes	Yes	3 hours before using data for RMR	Nights were excluded
39	r^2 threshold for slopes	Yes	Yes	between 0.7 and 0.95	between 0.7 and 0.85
40	Proportion of slopes removed	Yes	Yes		One individual had a R2 rejection % of 2.89%
41	When MMR measured	Not applicable	Yes		Before SMR
42	Method for MMR	Not applicable	Yes		Chase +1 min air exposure
43	Value taken as MMR	Not applicable	Yes		Highest oxygen uptake rate
44	Chase duration	Not applicable	Yes		70-131 seconds
45	Air exposure added	Not applicable	Yes		1-minute

46	Transfer time to chamber	Not applicable	Yes		<15 seconds
47	Duration of slopes for MMR	Not applicable	Not applicable		NA
48	Slope estimation method for MMR	Not applicable	Not applicable		NA
49	Aerobic scope calculation	Not applicable	yes		NA
50	Sample size	Yes	yes	4 pairs per year (8 fish/year) minus losses; see table 1	
51	How oxygen uptake was calculated	Yes	yes	Slope of DO decline during closed phase	Slope of DO decline during closed phase
52	Animal mass subtracted from volume?	Yes	Yes	Mass included in chamber-to-fish ratio	
53	Mass-correction/allometry	Partial	Yes	Missing masses replaced with yearly mean	

TABLE S2B. Checklist of 53 essential criteria for the reporting of methods for aquatic intermittent-flow respirometry for the laboratory respirometry.

Number	Criterion and Category	Response	Value (where required)	Units
	**EQUIPMENT, MATERIALS, AND SETUP**			
**1**	Body mass of animals at time of respirometry	Y	Table S2	
**2**	Volume of empty respirometers	Y	1.5 L and 3 L chambers	
**3**	How chamber mixing was achieved	Y	Tygon tubing + recirculation pump	

**4**	Ratio of net respirometer volume (plus any associated tubing in mixing circuit) to animal body mass	Y	41-56	
**5**	Material of tubing used in any mixing circuit	Y	Tygon	
**6**	Volume of tubing in any mixing circuit	Y	16 mL (1.5 L); 52 mL (3 L)	
**7**	Confirm volume of tubing in any mixing circuit was included in calculations of oxygen uptake	Y	Yes	
**8**	Material of respirometer (e.g. glass, acrylic, etc.)	Y	Glass	
**9**	Type of oxygen probe and data recording	Y	PyroScience Robust probe; FireSting-O2 + TEX4	
**10**	Sampling frequency of water dissolved oxygen	Y	~ every 2 s	
**11**	Describe placement of oxygen probe (in mixing circuit or directly in chamber)	Y	In recirculation loop	
**12**	Flow rate during flushing and recirculation, or confirm that chamber returned to normoxia during flushing	Y	4–5 L/min recirc; 10 L/min flush	
**13**	Timing of flush/closed cycles	Y	4-min flush; 7.5-min closed	
**14**	Wait (delay) time excluded from closed measurement cycles	Y	First 1.5 min excluded; slope over last 6 min	
**15**	Frequency and method of probe calibration (for both 0 and 100% calibrations)	Y	Before each experiment (0% & 100%)	Sodium sulphite was used to obtain a 0% solution for calibration.
**16**	State whether software temperature compensation was used during recording of water oxygen concentration	y	Yes (via TEX4)	
	**MEASUREMENT CONDITIONS**			
**17**	Temperature during respirometry	Y	6	°C

**18**	How temperature was controlled	Y	RTD probe + PID controller + mixing valve	
**19**	Photoperiod during respirometry	Y	Dark; red-light daily check	
**20**	If (and how) ambient water bath was cleaned and aerated during measurement of oxygen uptake (e.g. filtration, periodic or continuous water changes)	Y	Freshwater+chlorine; rinse; overnight seawater	
**21**	Total volume of ambient water bath and any associated reservoirs	Y	336	L
**22**	Minimum water oxygen dissolved oxygen reached during closed phases	Y	~80% for MMR, >95% for SMR	
**23**	State whether chambers were visually shielded from external disturbance	Y		
**24**	How many animals were measured during a given respirometry trial (i.e. how many animals were in the same water bath)	Y	3	
**25**	If multiple animals were measured simultaneously, state whether they were able to see each other during measurements	Y	No	
**26**	Duration of animal fasting before placement in respirometer	Y	≥72	hours
**27**	Duration of all trials combined (number of days to measure all animals in the study)	Y	4 days per trial	
**28**	Acclimation time to the laboratory (or time since capture for field studies) before respirometry measurements	Y	54	
	**BACKGROUND RESPIRATION**			
**29**	State whether background microbial respiration was measured and accounted for, and if so, method used (e.g. parallel measures with empty	Y	Empty chamber before & after	

	respirometry chamber, measurements before and after for all chambers while empty, both)			
**30**	State if background respiration was measured at beginning and/or end, state how many slopes and for what duration	Y	≥40 min before & after	
**31**	State how changes in background respiration were modelled over time (e.g. linear, exponential, parallel measures)	Y	Linear	
**32**	Level of background respiration (e.g. as a percentage of SMR)			
**33**	Method and frequency of system cleaning (e.g. system bleached between each trial, UV lamp)	Y	Freshwater + chlorine; overnight seawater between each trials	
	**STANDARD OR ROUTINE METABOLIC RATE**			
**34**	Acclimation time after transfer to chamber, or alternatively, time to reach beginning of metabolic rate measurements after introduction to chamber	Y	>24 h	
**35**	Time period, within a trial, over which oxygen uptake was measured (e.g. number of hours)	Y	~70	
**36**	Value taken as SMR/RMR (e.g. quantile, mean of lowest 10 percent, mean of all values)	Y	0.2 quantile, Chabot et al., 2016a	
**37**	Total number of slopes measured and used to derive metabolic rate (e.g. how much data were used to calculate quantiles)			
**38**	Whether any time periods were removed from calculations of SMR/RMR (e.g. data during acclimation, periods of high activity [e.g. daytime])	Y	Nights were excluded	
**39**	r^2 threshold for slopes used for SMR/RMR (or mean)	Y	Between 0.7 and 0.95	
**40**	Proportion of data removed due to being outliers below r-squared threshold	y	One individual had a R2 rejection % of 2.89%	

	**MAXIMUM METABOLIC RATE**			
**41**	When MMR was measured in relation to SMR (i.e. before or after)	Y	Before SMR	
**42**	Method used (e.g. critical swimming speed respirometry, swim to exhaustion in swim tunnel, or chase to exhaustion)	Y	Chase + 1-min air exposure	
**43**	Value taken as MMR (e.g. the highest rate of oxygen uptake value after transfer, average of highest values)	Y	Highest oxygen uptake rate	
**44**	If MMR measured post-exhaustion, length of activity challenge or chase (e.g. 2 min, until exhaustion, etc.)	Y	70–131	seconds
**45**	If MMR measured post-exhaustion, state whether further air-exposure was added after exercise	Y	1	minute
**46**	If MMR measured post-exhaustion, time until transfer to chamber after exhaustion or time to start of oxygen uptake recording	Y	<15	Seconds
**47**	Duration of slopes used to calculate MMR (e.g. 1 min, 5 min, etc.)	NA		
**48**	Slope estimation method for MMR (e.g. rolling regression, sequential discrete time frames)	NA		
**49**	How absolute aerobic scope and/or factorial aerobic scope is calculated (i.e. using raw SMR and MMR, allometrically mass-adjusted SMR and MMR, or allometrically mass-adjusting aerobic scope itself)	NA		
	**DATA HANDLING AND STATISTICS**			

**50**	Sample size	**Y**	Table 1	
**51**	How oxygen uptake rates were calculated (software or script, equation, units, etc.)	y	Linear decline during closed phase	
**52**	Confirm that volume (mass) of animal was subtracted from respirometer volume when calculating oxygen uptake rates	Y		
**53**	State whether analyses accounted for variation in body mass and describe any allometric mass-corrections or adjustments	Y		
